# The effects of yoga versus stretching and resistance training exercises on psychological distress for people with mild-to-moderate Parkinson’s disease: study prxotocol for a randomized controlled trial

**DOI:** 10.1186/s13063-017-2223-x

**Published:** 2017-11-02

**Authors:** JoJo Yan Yan Kwok, Jackie Cheuk Yin Kwan, Man Auyeung, Vincent Chung Tong Mok, Helen Yue Lai Chan

**Affiliations:** 1 The Nethersole School of Nursing, Faculty of Medicine, The Chinese University of Hong Kong, 6/F. Esther Lee Building, Shatin, Hong Kong, Special Administrative Region of China; 2 The Hong Kong Society for Rehabilitation, Lam Tin, Kowloon, Hong Kong, Special Administrative Region of China; 3 Department of Medicine, Pamela Youde Nethersole Eastern Hospital, Chai Wan, Hong Kong, Special Administrative Region of China; 4 Department of Medicine and Therapeutics, Therese Pei Fong Chow Research Center for Prevention of Dementia, Gerald Choa Neuroscience Centre, Faculty of Medicine, The Chinese University of Hong Kong, Shatin, Hong Kong, Special Administrative Region of China

**Keywords:** Parkinson’s disease, CAM, Mind-body intervention, Yoga, Mindfulness, Exercise, Anxiety, Depression, Randomized controlled trial, Study protocol

## Abstract

**Background:**

Psychological distress is prevalent among people with Parkinson’s disease (PD) and aggravates their motor symptoms, thereby leading to increased disability, high healthcare costs, and poor health-related quality of life (HRQoL). The under-recognition and adverse effects of the pharmacological management of anxiety and depression among the PD population are considerable. Thus, adopting a Complementary and Alternative Management (CAM) approach to address this problem is important. Yoga, one of the most common “mind-body” CAM therapies, can improve the psychological wellbeing of people with chronic illnesses. However, limited research on the effects of yoga in people with PD has been conducted. This study will determine the effects of yoga on the psychological wellbeing of people with mild-to-moderate PD and will compare these effects with those of stretching and resistance training exercises.

**Methods:**

A community-based, single-blind, randomized trial will be conducted. A total of 126 subjects will be recruited and randomly divided into yoga (*n* = 63) or stretching and resistance exercise (*n* = 63) groups. For 8 weeks, the yoga group will receive a weekly 90-min session of yoga, and the control group will receive a weekly 60-min session of stretching and resistance exercises. The primary outcome will be the level of psychological distress measured using the Hospital Anxiety and Depression Scale. The secondary outcomes will include the severity of motor symptoms measured by the Movement Disorders Society – Unified Parkinson’s Disease Scale − Part III Motor Examination; mobility, balance, and fall risk measured by the Timed Up and Go test; spiritual wellbeing measured by the Holistic Wellbeing Scale; and HRQoL measured by the Parkinson’s Disease Questionnaire-8. Assessment will be conducted at baseline, 8th, and 20th weeks of follow-ups.

**Discussion:**

This study will be the first randomized trial to compare the effect of yoga versus stretching and resistance training exercises in a PD population. Results will contribute to the value of yoga as a therapeutic option for managing psychological distress in PD patients. Multiple outcomes including psychological, physiological, and spiritual and HRQoL will also be measured to elucidate the potential mechanisms of yoga. The effect of yoga on people with chronic illnesses will further be elucidated. This information should contribute to future research, practice, and policy related to PD management.

**Trial registration:**

WHO Primary Registry – Chinese Clinical Trials Registry (ChiCTR): CUHK_CCRB00522 Registered on 8 October 2016; date of approval 19 August 2016.

**Electronic supplementary material:**

The online version of this article (doi:10.1186/s13063-017-2223-x) contains supplementary material, which is available to authorized users.

## Background

Parkinson’s disease (PD) affects approximately 10 million people worldwide and is the second most common chronic neurodegenerative disease. Based on demographic changes and prolonged life expectancy, the estimated prevalence of PD is expected to double by 2040 [1]. PD is characterized by four cardinal motor symptoms, namely, resting tremor, rigidity, bradykinesia, and postural instability. The management of PD primarily focuses on the pharmacological replacement of dopamine to control motor symptoms. However, nonmotor symptoms, such as anxiety and depression, reportedly have a great impact on health-related quality of life (HRQoL) and are thus gaining increasing attention [2].

Psychological distress, such as anxiety and depressive symptoms, are prevalent among individuals with PD. The incidence of anxiety and depression in this group is 27.6 to 56%, which is much higher than that in people with other chronic illnesses [3–9]. PD-affected individuals with psychological distress are found to be poor in social communication and prone to adopt a sedentary lifestyle, which compromises their daily role functions and self-care capacity, leading to early retirement and low HRQoL [10]. High psychological distress is also associated with increased disability, progression rate of physical symptoms, incidence of relapse, healthcare costs, and caregiver distress, as well as poor treatment compliance [11–13]. The devastating effects of psychological distress impair the functional status and HRQoL of PD patients, which greatly increase their need and cost for care.

Despite the high prevalence and substantial negative consequences of psychological distress in the PD population, this problem is poorly recognized and rarely addressed [6, 14]. The pharmacological management of anxiety and depression is complicated among PD patients; numerous potential drug interactions exist between antidepressants and PD treatments. Serotonergic agents may exacerbate PD symptoms. Bupropion has a potential dopamine agonistic effect benefitting PD symptoms but potentially worsening psychosis, whereas selegiline has antidepressant and anti-PD effects but may interact with levodopa and with other antidepressant agents [15]. Regarding the negative side effects of pharmacological treatments of anxiety and depression in PD patients, adopting a Complementary and Alternative Management (CAM) approach that addresses psychological distress in PD patients is important [16].

The beneficial effects of CAM on reducing anxiety and depressive symptoms have been demonstrated in individuals with chronic illnesses [17, 18]. Among all nonpharmacological CAM strategies, mind-body exercises are some of the most prevalent ones practiced by individuals with PD given their gentle and easy-to-follow nature [19]. A recent systematic review and meta-analysis of mind-body exercises, which include yoga, dancing, and Tai Chi, has revealed their immediate beneficial effects on the motor symptoms, postural instability, and functional mobility of individuals with mild-to-moderate PD [20]. Yoga has the largest beneficial effect on physical outcomes, but related research on psychological wellbeing is limited. To fill this research gap, this study will investigate the effects of yoga on the psychological wellbeing of individuals with PD.

### Conceptual framework

The Theory of Self-transcendence is the conceptual framework underpinning this research [21–23]. The purpose of this theory is to “provide a framework for inquiry and practice regarding promotion of wellbeing in the midst of difficult life situations, particularly where individuals and families face loss or life-limiting illness” (p.1) [23]. Self-transcendence is an underlying process that explains why people can attain wellbeing while confronting vulnerable conditions; it is theorized as a developmental capacity of a person that naturally emerges when a person encounters vulnerable health experiences. Self-transcendence involves the following four domains: (1) intrapersonal, i.e., how a person views situations that they are facing, (2) interpersonal, i.e., how people interact with others and the environment, (3) temporal, i.e., the capacity to integrate past experiences with outlook in life to obtain a meaningful view of the present situation, and (4) transpersonal, i.e., the capacity to connect with dimensions beyond the typically discernible world, also known as “spiritual consciousness.” How a person responds to difficult life situations, positively or negatively, affects the developmental process of a transcendent sense of self. If positively, people can “go beyond themselves” and self-transcend to attain a state of physical, psychosocial, spiritual wellbeing.

Yoga, which is known as “mindfulness in motion,” is a form of mind-body exercise promoting wellbeing of the body, mind, and spirit. Throughout the mindfulness practice of a sequence of poses (*asana*), breathing exercises (*pranayama*), and meditation (*dhyana*), the awareness and attention to body, mind, and the present moment is raised and sustained. The noncompetitive nature of yoga differs from many forms of exercises relying upon comparison to others or pushing oneself beyond limits to define progress. The guiding principle of yoga is to foster resilience for accepting adversities in life [24]. Through the regular mindfulness practice of yoga, a person can redefine the experience of pain, disability, or difficulty, thereby promoting one’s acceptance of disability and continual functioning beyond physical restraints and vulnerable conditions. Therefore, a yoga intervention is hypothesized to help a person with PD to explore one’s self-boundaries, redefine their illness experience, and self-transcend to attain a sense of wellbeing even in the vulnerable PD trajectory.

## Methods

### Aim

This study aims to examine the effect of yoga on the psychological wellbeing of individuals with mild-to-moderate PD. The objectives will be as follows: to investigate the effect of a structured mindfulness yoga program on (1) psychological wellbeing, (2) physiological wellbeing, (3) spiritual wellbeing, and (4) HRQoL in individuals with mild-to-moderate PD.

### Hypotheses

We hypothesize that (1) the structured mindfulness yoga program will be effective in improving psychological wellbeing, physiological wellbeing, spiritual wellbeing, and HRQoL in individuals with mild-to-moderate PD and (2) the structured mindfulness yoga program will be superior compared to stretching and resistance training exercise in improving psychological wellbeing, physiological wellbeing, spiritual wellbeing, and HRQoL in individuals with mild-to-moderate PD.

### Design

**Fig. 1:**
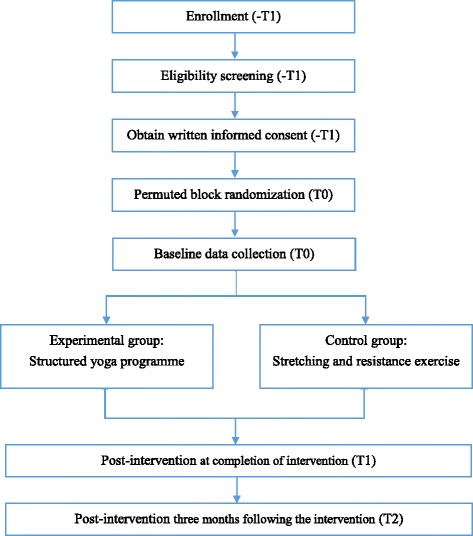
Consolidated Standards of Reporting Trials (CONSORT) flow diagram of the study

**Fig. 2:**
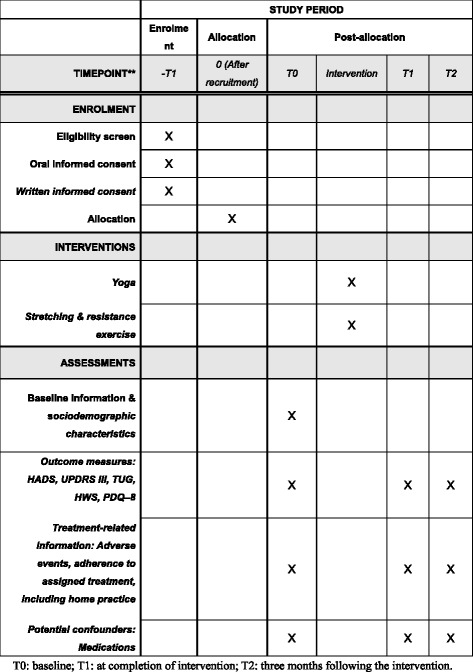
Standard Protocol Items: Recommendations for Interventional Trials (SPIRIT) Figure. The schedule of enrollment, interventions, and assessments. *T0* baseline, *T1* at completion of intervention, *T2* 3 months following the intervention

The study will be a single-blinded randomized controlled trial (RCT). Eligible participants will be randomized into experimental or control groups at 1:1 ratio through a permuted block randomization with a block size of 8 [25]. Figure 1 shows the Consolidated Standards of Reporting Trials (CONSORT) flow diagram, schedule of enrollment, interventions and assessments of the study, Fig. 2 shows the Standard Protocol Items: Recommendations for Interventional Trials (SPIRIT) Figure and Additional file 1 the SPIRIT Checklist utilized in the study.

### Study population

People with mild-to-moderate idiopathic PD will be recruited through two major sources: (1) two PD support groups and (2) two specialized outpatient clinics.

### Inclusion and exclusion criteria

Inclusion criteria include the following: (1) a clinical diagnosis of idiopathic PD, with a disease severity rating of stage I to III on the Hoehn and Yahr scale, (2) age above 18 years old, (3) ability to stand unaided and walk with or without an assistive device, and (4) participants who can give written consent. Exclusion criteria are the following: (1) participants who are currently receiving treatment for mental disorders or with uncontrolled mood disorders, (2) current participation in any other behavioral or pharmacological trial or instructor-led exercise program, (3) cognitive impairment as indicated by the abbreviated mental test score lower than 6, and (4) other debilitating conditions except PD, e.g., hearing or visual impairment, that can impede full participation in the study.

The inclusion criteria are developed to maximize the enrollment of appropriate participants, while the exclusion criteria are used to screen out patients who: (1) have secondary parkinsonism in which symptoms are caused by certain medications, a different nervous system disorder or another illness, (2) with debilitating medical conditions for which yoga or exercise is contraindicated, which pose a risk to safety, preclude fully informed consent, or hinder compliance with interventions (e.g., dementia), and (3) to minimize the risk of potential bias due to current or recent use of other behavioral or pharmacological interventions.

### Sample size

According to a meta-analysis of the effects of yoga on depression compared with aerobic exercise, a moderate effect size of 0.59 was reported for individuals with elevated levels of depression.

In addition, a moderate-to-large effect size of 0.79 on anxiety was found when comparing yoga with relaxation [26]. To anticipate the effect size of at least 0.59 for our primary outcomes (depression and anxiety) and using the power analysis software Gpower 3.1, 47 subjects per arm give a two-arm RCT 80% power to detect an effect size of at least 0.59 at 5% level of significance. Considering previous RCTs of mind-body interventions among people with PD [20], an attrition rate of 25% is taken into consideration. Therefore, 126 subjects with 63 per arm will be recruited.

### Sampling and recruitment strategies

Convenience sampling will be used. Potential study participants will be recruited through PD support groups and outpatient clinics. PD support groups will include the following: (1) the Hong Kong Society for Rehabilitation, a nonprofit rehabilitation organization serving over 100,000 members through its own primary-care facilities in Hong Kong and (2) Hong Kong Parkinson’s Disease Association, a PD patient support association serving over 1200 members.

Combinations of advertising strategies that have been found to be successful in prior recruitment initiatives for PD trials will be adopted [27–29]. These strategies will include printed flyers within the patient support centers and neurological clinics. Information will be disseminated through newsletters of patient support groups, which are expected to reach around 100,000 members. Enrollment will be further aided by collaboration with outpatient clinics, including the Pamela Youde Nethersole Eastern Hospital and the Prince of Wales Hospital. Potential participants will be referred by neurologists during their regular medical follow-up visits. Information and registration will be accessible online.

### Interventions

#### Experimental group

Participants in the experimental group will attend an 8-week structured yoga program. Each yoga session will last for 60 min and will be conducted in a group format once a week. There will be around 15–20 participants for each class to provide adequate instructional attention to each participant and to allow the yoga instructor to perform the required training routines. Participants will be encouraged to perform a 20-min, home-based practice twice a week (Table 1: yoga protocol).

**Table 1:** Overview of the mindfulness yoga intervention

Week	1 − 2	3 − 4	5 − 6	7 − 8
Theme	Introducing mindfulness yoga to our body and life	Mindfulness of the body and content of mind		Loving-kindness and compassion
Breathing exercise (15 min)	Controlled breathing			
	Bee breath			
	Lion breath			
	Cooling breath			
	Alternate nostril breathing			
Mindfulness practice (15 min)	Mindfulness of breath and body meditation (body scan)	Mindfulness of body and thoughts meditation (body scan)	Mindfulness of feelings and movement meditation (mindful walking)	Loving-kindness and open awareness meditation (dyad practice)
Yoga practice (60 min)	Warm-up exercise (15 min)			
	Child pose			
	Cat and cow			
	Bridge			
	Lying hamstring stretch			
	12 poses of “sun salutation” with modifications (30 min)			
	Mountain pose			
	Upward salute pose			
	Standing forward bend			
	Lunge			
	Plank pose			
	Knees, chest, and chin pose			
	Cobra pose			
	Downward-facing dog pose			
	Lunge			
	Standing forward bend			
	Upward salute pose			
	Mountain pose			
	Cool-down exercise (15 min)			
	Child pose			
	Knee to chest			
	Corpse pose			
	Easy pose			

The dose of the yoga intervention took reference from the remarkable Mindfulness-based Stress Reduction (MBSR) program [30]. MBSR, which consists of eight weekly mindful yoga sessions, has been shown to be an effective behavioral intervention for people with stress, anxiety disorders, and chronic illness [31–33].

Twelve basic *Hatha* yoga poses, known as “sun salutations” (*surya namaskar*), with controlled breathing (*pranayama*) and mindfulness meditation (*dhyana*) will be taught. This set of yoga motions and postures is a gentle yet dynamic form of exercise that involves static stretching while exerting optimal stress on the cardiorespiratory system, and it has been described as the most complete yoga exercise [34]. During the mindfulness practice of yoga poses, participants will be taught to control their breathing and to envisage vital energy flowing freely between the body and mind, forming a deep connectedness to mind and spirit, reaching a state of equanimity.

#### Control group

To enhance the internal validity of the study, the participants in the control group will undergo weekly, 60-min stretching and resistance exercise and be encouraged to perform a 20-min, home-based practice twice a week for 8 weeks (Table 2: stretching and resistance exercise protocol). Current evidence shows that both stretching and resistance exercises and yoga improve motor symptoms and functions in the PD population [19, 35]. Since the former only focused on physical wellbeing, it has been chosen for the control group. The set of stretching and resistance exercises was modified from a previously validated study for older adults with osteoarthritis [36]. A fitness instructor will be recruited to deliver the intervention. These regular gatherings will be aimed at balancing the psychosocial effect of the weekly gathering of the experimental groups during the study period. No information relating to yoga, mindfulness meditation, and psychological wellbeing will be covered in stretching and resistance exercise classes. One class will have approximately 15–20 participants.

**Table 2:** Overview of the stretching and resistance exercise intervention

Week	Warm up	Resistance training	Stretching and cool down
1 − 2	Seated: Straight arm forward rotation^1^ Straight arm backward rotation^1^ Straight arm up-and-down flapping^1^ Seated forward and backward stepping^1^ Seated hip abduction and adduction stepping^1^ Seated knee raise^1^	Seated: Seated forward leg extension^1^ Seated backward leg curl^1^ Standing^a^: Single leg knee raise^2^ Alternate knee raise^1^ Mini squat (30°)^1^	Seated: Seated hamstring stretch^2^
3 − 4	Seated: Straight arm forward rotation^1^ Straight arm backward rotation^1^ Straight arm up-and-down flapping^1^ Straight arm horizontal abduction and adduction^1^ Trunk rotation and hold^1^ Standing^a^: Straight leg forward kick^1^ Straight leg backward kick^1^ Straight leg hip adduction and abduction^1^ Alternate knee raise^1^	Seated: Straight leg forward kick^2^ Straight leg circling^2^ Standing^a^: Calf raise^2^ Mini squat (45°)^2^	Standing^a^: Hamstring stretch^2^ Calf stretch^2^
5 − 6	Seated: Straight arm forward rotation^1^ Straight arm backward rotation^1^ Straight arm up-and-down flapping^1^ Straight arm horizontal abduction and adduction^1^ Trunk rotation and hold^1^ Standing^a^: Straight leg forward kick^1^ Straight leg backward kick^1^ Straight leg hip adduction and abduction^1^ Alternate knee raise^1^	Seated: Straight leg circling^2^ Straight leg alternate crossover^2^ Standing^a^: Single leg standing^2^ Single leg calf raise^2^ Wall squat^2^	Standing^a^: Hamstring stretch^2^ Calf stretch^2^ Upper trapezius stretch^2^
7 − 8	Seated: Straight arm forward rotation^1^ Straight arm backward rotation^1^ Straight arm up-and-down flapping^1^ Straight arm horizontal abduction and adduction^1^ Trunk rotation and hold^1^ Standing^a^: Straight leg forward kick^1^ Straight leg backward kick^1^ Straight leg hip adduction and abduction^1^ Alternate knee raise^1^	Standing^a^: Single leg standing^2^ Single leg calf raise^2^ Side lunges^2^ Wall squat^2^	Standing^a^: Hamstring stretch^2^ Calf stretch^2^ Upper trapezius stretch^2^ Side trunk flexion stretch^2^ Forearm stretch^2^

^a^Standing with hands rest on chair back; ^1^10 s, two cycles, with 10 s rest; ^2^20 s, two cycles, with 10 s rest

### Validity and reliability

#### Content validity

To assure the validity of the novel mindfulness yoga intervention, including appropriateness, safety, and applicability, the yoga protocol was reviewed by a representative panel of seven experts with clinical expertise, including one neurologist, one PD nurse specialist, one physical education researcher specialized in balance and fall, one physiotherapist, one occupational therapist, and two yoga instructors, both of whom are experienced in teaching people with chronic illnesses and one of them is a certificated “Mindfulness-based Cognitive Therapy” (MBCT) and “Mindfulness-based Stress Reduction” instructor.

The yoga protocol has been validated. The content validity of each items of intervention was estimated by the Item-Content Validity Index (I-CVI), which guided the decisions of item revisions or rejections. An I-CVI of 0.78 or higher was considered good content validity [37]. Meanwhile, comments from experts guided the development of any new item and amendment of existing items.

Two rounds of panel review were conducted. During the first round, among the 20 poses, the following modifications were made according to expert’s opinions:

1. The axial rotation in reclined spinal twist pose was considered as less relevant to be one of the warm-up exercises of the yoga practice (sun salutations). This pose was replaced by the child pose, which can gently stretch the shoulders, neck, back, hips, thighs, and ankles
2. Standing forward bend and downward-facing dog poses might be too difficult for participants with postural instability. Therefore, these two poses were modified as keeping the feet hip-distance wide and bending the knees to make it easier for those beginners or participants with postural instability
3. An inquiry was raised to determine as to whether the corpse pose was a kind of meditation. Actually, it is a common cool-down exercise of yoga, which positions the body in a neutral position of rest and relaxation. Thus, this pose remained unchanged with the purpose of enhancing one’s mindful awareness of breath, body, and mind

In the second round, the I-CVIs of all poses reached 1.0. The finalized protocol of the yoga intervention is listed in Table 1.

#### Randomization and allocation concealment

Eligible participants will be randomized into the experimental or the control group with an allocation of 1:1 by using a computer-based permuted block randomization with a block size of 8. The randomization sequence will be generated by an independent research assistant. Details of the group allocation will be concealed on cards inside a sequentially numbered series of sealed opaque envelopes. The envelopes will then be submitted to a research coordinator responsible for contacting participants and allocating them to their assigned group.

#### Blinding

This will be a single-blind RCT. The outcome assessor will be blinded to the subject allocation. The outcome assessor, who is independent from group allocation, will be responsible for outcome measurement. However, it would be hard to blind the participants given the nature of the intervention. This single-blind design may potentially expose the study to risks of bias in terms of performance, attrition, and evaluation and so inflate the treatment effects if the participants know their group allocation. Although blinding the participants to intervention assignment will be inapplicable in this study, various strategies will be used to minimize these risks. To minimize bias stemming from expectation, study participants will be blinded to the study hypotheses and details of study manuals, the information provided in the Informed Consent Form will follow the principle of equipoise by declaring uncertainty about the superiority of treatment effect of both arms [38]. Similar expected outcomes, such as improving general wellbeing, will be stated in the Information Sheet for both experimental and control groups. All participants will receive the same number of sessions of intervention and assessment. The participants will be reminded not to disclose their group status to the study assessor at any time point. This method is widely used to mask the outcome assessor from treatment assignment as recommended by the Evidence-based Behavioral Medicine Committee [39].

#### Attrition and missing data

Regarding attrition and missing data, an attrition rate of 25% is considered in this trial, and we will monitor the progress of the trial with respect to this target. To reduce attrition and missing data, the importance of full and complete participation in the trial, regardless of the treatment assignment, will be conveyed to the study team members and participants. Follow-ups will be arranged at participants’ convenience to minimize the burden of data collection. Participants will be contacted on telephone for follow-up arrangement, and two follow-up reminders will be delivered to participants, via telephone call or WhatsApp message at 2 weeks, and 1 day before follow-up. In order to facilitate a positive study experience among participants, all participants will be attentively followed up. All study procedures will be described clearly to the participants, and their performance assessment and its corresponding health implications will be explained individually at the end of each follow-up by the assessor. Contact information of participants will be kept up to date and reasons of dropout will be documented [40].

#### Adherence

All participants will be instructed to follow their normal medication schedule and physical activity and not to start any new instructor-led exercise program throughout the study period. All classes will be held at local community facilities. Picture-based education booklets with enlarged picture guides will be provided to participants to facilitate home-based practice (see Additional file 2: Yoga Educational Booklet). All participants will also be instructed to perform a 20-min, home-based practice twice a week. A self-reported activity log will be provided to monitor participants’ adherence to home-based practice. Class attendance will be checked by the instructor. Reasons for absenteeism will be also documented and verified by the research coordinator.

### Outcomes

#### Primary outcome

Psychological wellbeing in terms of anxiety and depression will be measured by the Hospital Anxiety and Depression Scale (HADS) [41, 42]. This scale has been suggested for use in the PD population as somatic symptoms that may potentially overlap parkinsonian manifestations are not assessed in this scale [43, 44]. The HADS consists of two subscales, namely, anxiety and depressive symptoms. Each subscale consists of seven items, and each item is rated by a four-point scale (range: 0–3). Levels of symptoms of anxiety and depression are considered clinically relevant at a cutoff value of ≥ 8 on each subscale and ≥ 15 for the full scale. The Chinese version of the HADS has demonstrated satisfactory validity and reliability with a Cronbach’s alpha of 0.86 for the overall scale, 0.82 for the depression subscale and 0.77 for the anxiety subscale [41, 42].

#### Secondary outcomes

Secondary outcome measures will include the following: (1) motor symptoms, (2) mobility, balance, and fall risk, (3) spiritual wellbeing, and (4) HRQoL. Details of each outcome and instrument are as follows:

1. *Motor symptoms*
  Movement Disorders Society – Unified Parkinson’s Disease Rating Scale – Part III Motor Examination (MDS-UPDRS-III) is an 18-item, assessor-rated instrument assessing severity of PD motor symptoms. Each item is scored “0” to “4” on a categorical scale, in which “0” indicates no impairment and “4” represents severe impairment. The total score is 132. Higher scores indicate more motor disabilities. This scale has demonstrated excellent internal consistency with a Cronbach’s alpha of 0.93. Excellent concurrent validity has been demonstrated by the good correlation with the original UPDRS (*r* = 0.96, *p* < 0.001) [45]
2. *Mobility, balance, and fall risk*
  The Timed Up and Go test (TUG) is a simple test used to assess mobility, balance, and fall risk in older adults. It is used to count the time required for a participant to rise from a chair, walk for 3 m, turn around, walk back to the chair and sit down. Satisfactory test-retest reliability has been demonstrated in the PD population with intracluster correlation coefficient (ICC) ranging from 0.80 to 0.85 [46, 47]. Excellent concurrent validity has been demonstrated by good correlation with the Berg Balance Scale (– *r* = 0.78, *p* < 0.001) [48]. The minimal detectable change for the Chinese population with PD was 3.5 s [46]. The cutoff score for fall prediction among PD is 11.5 s with a sensitivity of 0.66 and specificity of 0.62 [35]
3. *Spiritual wellbeing*
  The Holistic Well-being Scale (HWS) is a 30-item questionnaire that evaluate the two domains of holistic wellbeing, as follows: (1) the absence of affliction characterized by bodily irritability, emotional vulnerability, and spiritual disorientation and (2) the presence of equanimity in terms of general vitality, mindful awareness, nonattachment, and spiritual self-care. Each item is rated using a “0–10” scale, where “0” represents totally disagree, whereas “10” indicates totally agree. Higher scores indicate a worse state of holistic wellbeing. Satisfactory psychometric properties has been demonstrated in the Chinese version [49]
4. *HRQoL*
  The Parkinson’s Disease Questionnaire-8 (PDQ-8) is a short-form, 8-item questionnaire used to quantify the impact of PD on the HRQoL of patients [50]. This questionnaire assesses eight domains, as follows: mobility, activities of daily living, emotional wellbeing, stigmatization, social support, cognitions, communication, and bodily discomfort. A PDQ-8 summary index is generated. Higher scores indicate worse HRQoL. The internal consistency of the Chinese version of PDQ-8 has been validated with a Cronbach’s alpha of 0.87, and construct validity has been demonstrated against the Hoehn and Yahr scale and UPDRS motor scores [51]

### Data collection

The principal investigator will screen the eligibility of potential participants. For these eligible individuals, an Information Sheet of the study will be provided, and written informed consent will be sought. Information related to demographics and health background will then be collected (Appendix 3). All primary and secondary outcomes will be measured at baseline (T0), 2 months (T1; completion of intervention), and 3 months following the completion of the intervention (T2). All assessment will be conducted during the “on” state of medications to minimize motor fluctuations among participants. Participants will be instructed to follow their normal medication schedule and physical activity and not to start any new, instructor-led exercise program throughout the study period.

### Data analysis

Descriptive statistics will be used to summarize the demographic, health conditions and clinical outcomes of the participants at three different time points. Normality of the variables will be assessed using the skewness statistic and normal probability plot. The two treatment groups will be examined, at baseline (T0), after the completion of intervention (T1), and 3 months after the completion of intervention (T2), for the changes in psychological distress, motor symptoms, mobility and balance, spiritual wellbeing, and HRQoL among participants. The intention-to-treat principle will be applied in all analyses. The intervention arm will be compared against the control for all primary analysis. Generalized estimating equation (GEE) models, specific with first-order, auto-regressive structure, will be used to assess the differential change of each primary outcome variable (HADS) and secondary outcome variable (MDS-UPDRS-III, TUG, HWS, and PDQ-8), across different time points between the two groups. Completers and noncompleters will be compared to examine any difference in demographic characteristics and health conditions. Prespecified subgroup analyses by gender, level of education, disease staging, and the presence of psychological distress will be conducted. IBM SPSS 22.0 software will be used for statistical analysis. All statistical tests will be two-tailed and statistical significance will be set at 0.05. The final report will follow the CONSORT 2010 guidelines as well as its extension to nonpharmacological interventions.

### Ethical considerations

#### Autonomy, privacy, and confidentiality

Ethics approval was obtained from The Joint Chinese University of Hong Kong – New Territories East Cluster Clinical Research Ethics Committee (2016.323–T) in August 2016. The study was registered in the WHO Primary Registry – Chinese Clinical Trials Registry (ChiCTR) (CUHK_CCRB00522) in August 2016. Written informed consent will be obtained from eligible participants before any assessment or intervention. The participation will be voluntary and participants will be allowed to withdraw from the study at any time point. Confidentially will be maintained using unique identification numbers of subjects. All data will be kept in a locked and secured cabinet, and access will be restricted to the research staff. This protocol is stated in compliance with the Declaration of Helsinki.

#### Risks and safety

Yoga is generally regarded as safe, and adverse events are rare. The major potential risks of yoga are muscle strains or injuries. Therefore, adequate warm-up and cool-down exercises are emphasized in this program. Props, such as towel, chair, and wall, will be used to support participants toward some more challenging poses. Apart from that, each posture of this yoga program has been tailor-made and reviewed by the expert panel to ensure that it will be delivered in a progressive and safe manner for individuals with mild-to-moderate PD. The interveners of both arms will have at least 2 years of teaching experience among individuals with chronic illnesses. In addition, clinical trial insurance will be purchased for all participants in the experimental group. Participants will be instructed to report any unexpected or unusual symptom to the principal investigator. A project adverse log will be used to document any adverse events. Any major serious adverse event will be reported immediately to the Ethics Committee. No formal stopping rules will be established in advance for efficacy. The trial will be stopped only if there is an unacceptable risk of serious adverse events in one or both of the treatment arms. In this case, investigators will recommend terminating either or both of the arms of the entire study.

### Pilot study

A pilot study of the yoga intervention was conducted from September to November 2016 [52]. Ten people with mild-to-moderate idiopathic PD were recruited with the same inclusion and exclusion criteria. Assessments were done before and after completion of this 8-weekly, 60-min yoga program to evaluate the feasibility of the yoga program. The enrollment rate was 58.8% and class adherence rate was 97.5%. There were no adverse events. Results of process evaluation are summarized in Table 3. All participants were satisfied with this program. Perceived benefits included improving flexibility, gait and balance, and reducing constipation, lessening back and shoulder pain, relaxing body and mind, increased ease of falling asleep, less anxiety, increased calmness, and better mood. From the preliminary data analysis, significant improvements were found for (1) psychological distress in terms of the HADS *(paired t test (T*_*1*_ *–T*_*0*_ *): mean = – 4.7 ± 4.16, p = 0.006)* and (2) holistic wellbeing in terms of affliction *(paired t test (T*_*1*_ *–T*_*0*_ *): mean = – 0.87 ± 0.80, p = 0.007)*. These findings support the hypothesis that yoga intervention can help PD individuals to improve their psychological wellbeing and change their perception of affliction.

**Table 3:** Summary of process evaluation of pilot study (*n* = 10)

%	Strongly agree	Agree	No comment	Disagree	Strongly disagree
1. The content of exercise program is appropriate	60	30	10		
2. The total number of classes and duration of each class was appropriate − too short for each class (*n* = 1)	30	60		10	
3. The venue was appropriate	30	70			
4. I think that the difficulty level of exercise being taught in classes is appropriate for me	40	50	10		
5. I think that the breathing exercises are useful for me	40	60			
6. I think that the mindfulness meditation is useful for me	30	60	10		
7. I think that the yoga poses are useful for me	50	50			
8. The information being provided related to this exercise program is appropriate	40	50	10		
9. I am satisfied with the teaching methods delivered by the instructor	80	20			
10. I can remember the exercises learned from class	30	50	10	10	
11. In daily living, I have time to practice the exercises learned from class	30	60	10		
12. After the completion of exercise program, I am confident that I could continue the exercise practice in daily living	30	40	20	10	
13. Speaking overall, I am satisfied with this exercise program	50	50			

From the pilot study, the yoga intervention and study procedures were found to be feasible and acceptable for people with mild-to-moderate PD, except that the instructor proposed having more time for mindful practice in each session. Hence, the duration of each class is increased from 60 min to 90 min, so as to ensure adequate time for participants to immerse in mindfulness practice in a gradual manner.

## Discussion

The findings from this study will contribute to knowledge generated from previous research that has demonstrated the benefits of yoga in terms of improving physical wellbeing, such as motor symptoms, postural instability, and functional mobility. This study will broaden the body of research on the use of yoga to address psychological distress among people with chronic illnesses, including neurodegenerative conditions. The minimal side effects and low cost of yoga intervention, compared with other current treatments, make it attractive for populations who are expected to experience repetitive and progressive disequilibrium resulting from degenerating PD conditions while remaining active in daily and recreational activities. The mindfulness yoga program, if found effective for alleviating psychological distress, decreasing motor symptoms, and improving physical wellbeing and HRQoL, can serve as a low-cost community-based and self-help tool for long-term practice among PD patients.

Research on yoga as a therapeutic modality is relatively new. Given the complexity and heterogeneity of yoga practices, structured protocols that are evidence based and reproducible are crucial if widely disseminated. Future research investigating yoga interventions among people with chronic illnesses can adopt and utilize this validated yoga protocol. Another focus of this study will be the implementation and evaluation of this evidence-based yoga intervention. The feasibility, acceptance, and practicability of a mindfulness yoga program for PD patients will be evaluated. This research will also estimate the attrition rates, response rates, variability in outcomes, willingness to accept randomization, and potential sampling biases in PD population.

Our results will influence the current approaches to managing psychological distress, such as anxiety and depression, in the PD population. This approach has a more integrative, interdisciplinary focus on active self-care approaches that will empower patients to achieve a mindful awareness of their condition, thereby guiding them to participate in the self-transcendence process to reach a sense of wellbeing while handling the vulnerable illness trajectory.

### Limitations

We acknowledge two major limitations of this study. First, participation to the study is on a voluntary basis and limit to the mild-to-moderate stage only. People with PD who experience depressive and anxiety symptoms may be less likely to participate in the study. Also, people who are currently taking psychiatric treatment will be excluded. This may limit the generalizability of the findings to those with significant psychological distress. To ameliorate the effects of participation bias, subject recruitment will be conducted in both support groups and outpatient clinics in different geographical areas to increase access to a wider range of people with PD. Second, the single-blind design may expose the study to risks of bias stemming from performance, attrition, and evaluation, which may overestimate the treatment effects. Strategies for minimizing these risks are discussed previously.

### Trial status

This study is going to be commenced in March 2017. Estimated trial completion is expected by March 2018.

## Additional files

Additional file 1: SPIRIT Checklist. (DOC 120 kb)

Additional file 2: Yoga Educational Booklet. (DOCX 2629 kb)

## Abbreviations

CAM: Complementary and Alternative Management
ChiCTR: Chinese Clinical Trials Registry
GEE: Generalized estimating equation
HADS: Hospital Anxiety and Depression Scale
HRQoL: Health-related quality of life
HWS: Holistic Wellbeing Scale
I-CVI: Item-Content Validity Index
ITT: Intention-to-treat
MBCT: Mindfulness-based Cognitive Therapy
MBSR: Mindfulness-based Stress Reduction
MDS-UPDRS-III: Movement Disorders Society – Unified Parkinson’s Disease Rating Scale – Part III Motor Examination
PD: Parkinson’s disease
PDQ-8: Parkinson’s Disease Questionnaire-8
RCT: Randomized controlled trial
TUG: Timed Up and Go test
