# Supplementary material for: The effects of yoga versus stretching and resistance training exercises on psychological distress for people with mild-to-moderate Parkinson’s disease: study prxotocol for a randomized controlled trial
Source: Trials. 2017 Nov 2;18:509. doi: 10.1186/s13063-017-2223-x (PMC5667474; doi:10.1186/s13063-017-2223-x)
Supplement: Supplementary file 2 — Yoga Educational Booklet. (DOCX 2629 kb) [file 13063_2017_2223_MOESM2_ESM.docx]

**
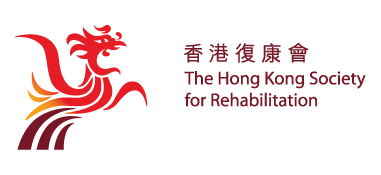

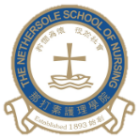

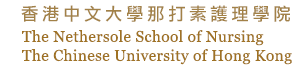

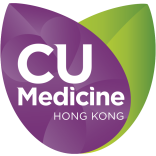

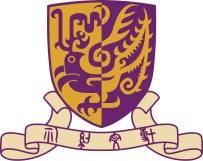
**

**柏金遜症正念瑜伽課程**

瑜伽，意指「聯繫、結合、統一」。廣義來說，是一種強調身體、心靈與精神和諧統一的運動方式。本課程為期八節，內容包括調身的正念瑜伽體位練習、調息的呼吸練習和調心的靜觀練習，強調透過身體覺察來放鬆身體繃緊的位置，並透過瑜伽動作練習來提昇身體機能和靈活性，促進患者的身心靈健康。

此課程的內容如下:

| 星期 | **1-2** | **3-4** | **5-6** | **7-8** |
| --- | --- | --- | --- | --- |
| 主題 | 日常生活中的正念 | 身心的正念 | | 慈心的正念 |
| 呼吸練習 (15分鐘) | - 蜂鳴呼吸法 - 獅子呼吸法 - 清涼呼吸法 - 左右鼻孔呼吸法 | | | |
| 正念瑜伽體位練習 (60分鐘) | 熱身運動　(15 分鐘)   - 嬰兒式 - 貓牛式 - 橋式 - 提腿腳踝伸展 | | | |
|  | 拜日十二式　(30 分鐘) | | | |
|  | 伸展運動　(15 分鐘)   - 嬰兒式 - 平躺抱膝 - 簡易坐 - 大休息 | | | |
| 靜觀練習 (15分鐘) | 覺察呼吸練習 | 身體描掃練習 | 靜觀步行練習 | 慈心靜觀練習  雙人互助動作練習 |

**呼吸練習**

瑜伽中的「調息法」（Pranayama）即是呼吸練習，是一系列有規律、有意識的吸氣、呼氣和屏息練習，亦被視為實踐瑜伽的源頭。呼吸的方式與人的身心狀態有着本質的聯繫，心境平和時呼吸緩慢深長，緊張憤怒時則短淺急促。正確的呼吸練習能增加肺活量，提供血液更多的氧氣，使人精力充沛。透過平穩而覺察的呼吸練習，為散亂的心導入專注，平復情緒的波動，讓人感到平靜，讓身心得以休憩。

**蜂鳴呼吸法**

1.
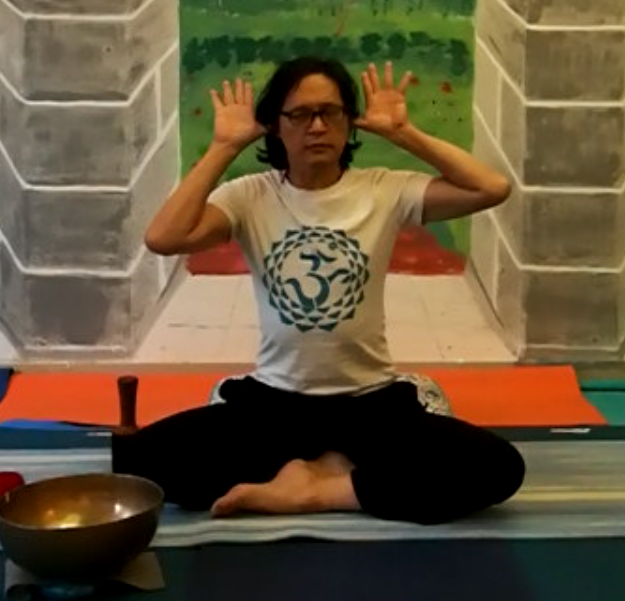
採取舒適的坐姿，雙手放在雙膝，保持頭、頸、背挺直。閉上雙眼，放鬆全身片刻。
2. 輕輕閉上嘴，牙齒略微分開一點，用雙手食指堵住雙耳，身體保持穩定。
3. 用鼻子深吸氣，然後緩慢呼氣，同時發出平穩像蜜蜂鳴叫般的嗡嗡聲。呼氣時保持流暢連貫、柔和不間斷的蜂鳴聲，前額會感覺到蜂鳴的迴音，集中意念於聲音的振動上面。

**獅子呼吸法**

1.
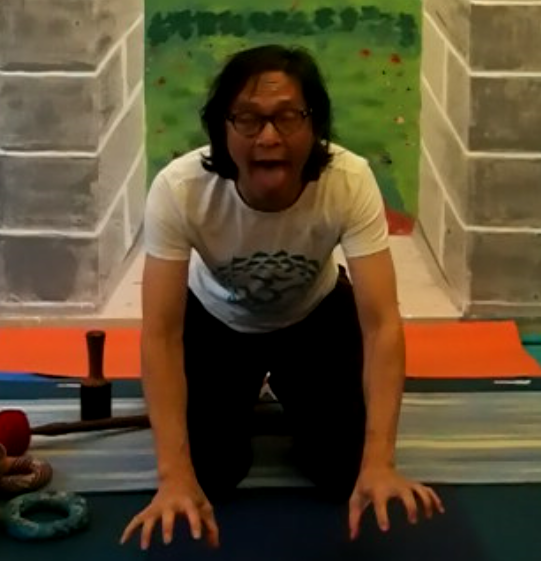
採取跪姿＊，雙腳膝蓋稍微打開，掌心朝下放在大腿上，腳趾可以撐地或平放地上。
2. 用鼻子深吸氣，然後將肩膀稍微抬高，雙臂伸直。
3. 像獅子吼般大叫一聲「啊」來吐氣，嘴巴大開來伸展你的舌頭，並將舌頭向下伸向下巴，眼睛盡量張大向上望，掌心用力下壓，手指張開。
   - 患有膝蓋或腳踝疾病的人，不建議採取跪姿，可以舒適坐姿代替。

**清涼呼吸法**

1.
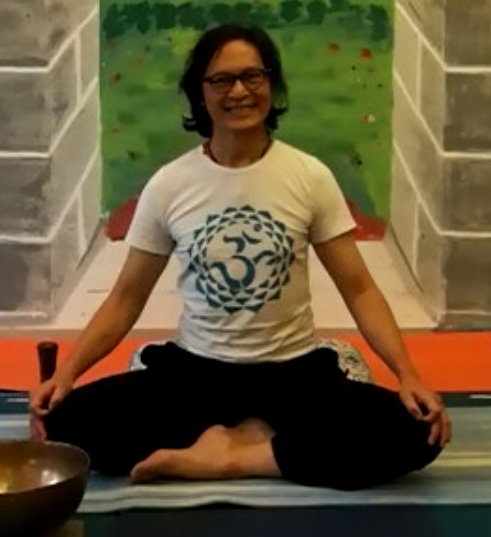
採取舒適的坐姿，雙手放在雙膝，保持頭、頸、背挺直。閉上雙眼，放鬆全身片刻。
2. 舌頭前伸，觸及牙齒內側，嘴嘴唇微張，上下齒之間留有縫隙，讓空氣可以從縫隙中進入口中。
3. 用嘴慢慢吸氣，能夠聽到和感到清涼的空氣通過口腔慢慢進入；接著慢慢通過鼻孔呼出空氣，整個過程都採用完全式呼吸。

**左右鼻孔呼吸法**

1.
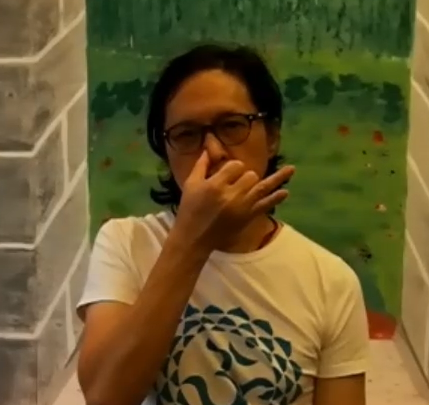
採取舒適的坐姿，抬起右手，彎曲食指和中指，把剩下的大拇指、無名指和小指伸直。
2. 先用大拇指按住右鼻孔，用左鼻孔吸氣。之後用無名指和小拇指閉住左鼻孔，由右鼻孔呼氣，然後繼續由右鼻孔吸氣，再用大拇指閉住右鼻孔，鬆開左鼻孔，由左鼻孔呼氣，此為一個呼吸回合。
3. 練習時吸氣和呼氣的時間均等，重覆這組動作。在整個練習中，不應有呼氣急促的感覺，吸入量以呼出時不費力為限；舒適前提下，再逐漸增加呼吸的時間。

呼吸練習注意事項：

- 每個呼吸練習可重複三至五次，每五分鐘的練習應休息一分鐘，總呼吸練習的時間為十五分鐘。
- 呼吸衰竭、感染未受控制或臨床情況不穩定者需醫生許可方可練習。
- 練習時應恰當掌握呼吸的節奏和時間，保持緩慢而深長、放鬆的深呼吸。練習時切忌過於頻繁及用力，以避免因過於淺快的呼吸而引起的過度換氣綜合症。如有頭暈、胸悶、呼吸不穩定的徵狀應立即停止練習。

**正念瑜伽體位練習**

- 最佳的運動時間是在自己活動能力良好的時候，通常在服用柏金遜藥物後的一小時。每人對藥物的反應都有所不同，因此你可以根據自己的最佳的活動時間決定和安排自我練習。
- 做瑜伽體位練習前僅記先做至少十五分鐘的熱身運動。熱身運動能促使人體從平常安靜的狀態，過渡至正式運動時較為緊張的肌肉活動狀態，增進肌肉反應靈活，從而減少運動傷害。
- 練習過後僅記做至少十五分鐘的緩和運動。緩和運動能讓人的心跳在運動過後逐漸下降至正常水平，放鬆肌肉，並讓身體回復正常的狀況。若出現頭昏眼花或嗜睡的情況，建議柏友用較長的時間進行緩和運動。
- 每當我們練習的時候，都要集中意念，保持緩慢深長的呼吸，完全地放鬆身心，讓身體的緊張感覺隨著動作慢慢消失。謹記，每人身體的拉伸極限和柔韌性都是不同的，不要強迫、不要過度彎曲身體，只要做到你能做到的即可。若有不適的感覺，請停止練習。

**熱身運動**


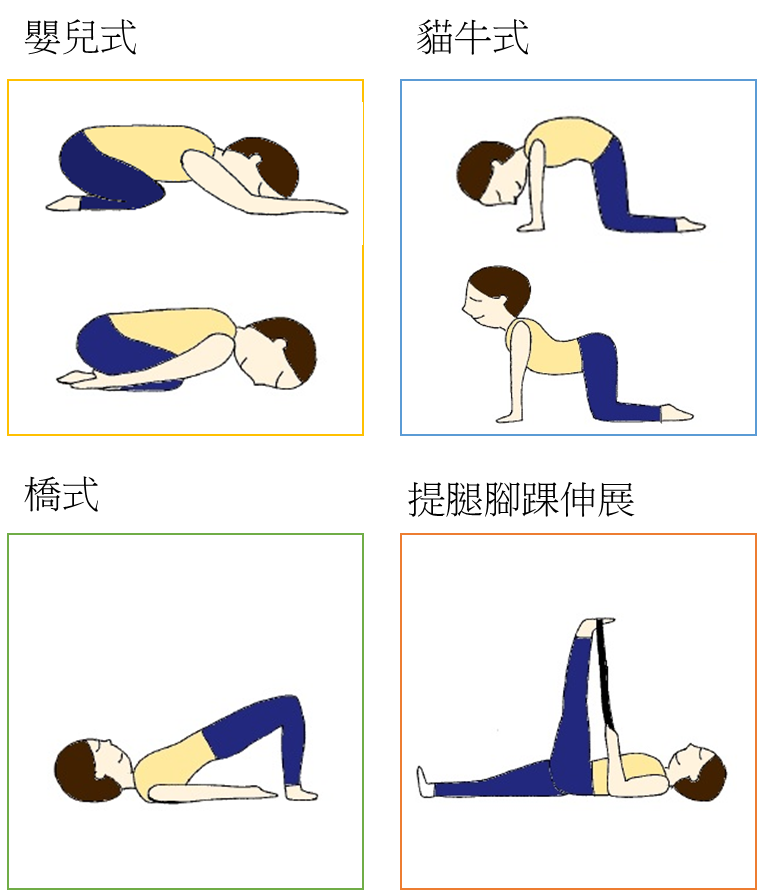


**拜日十二式
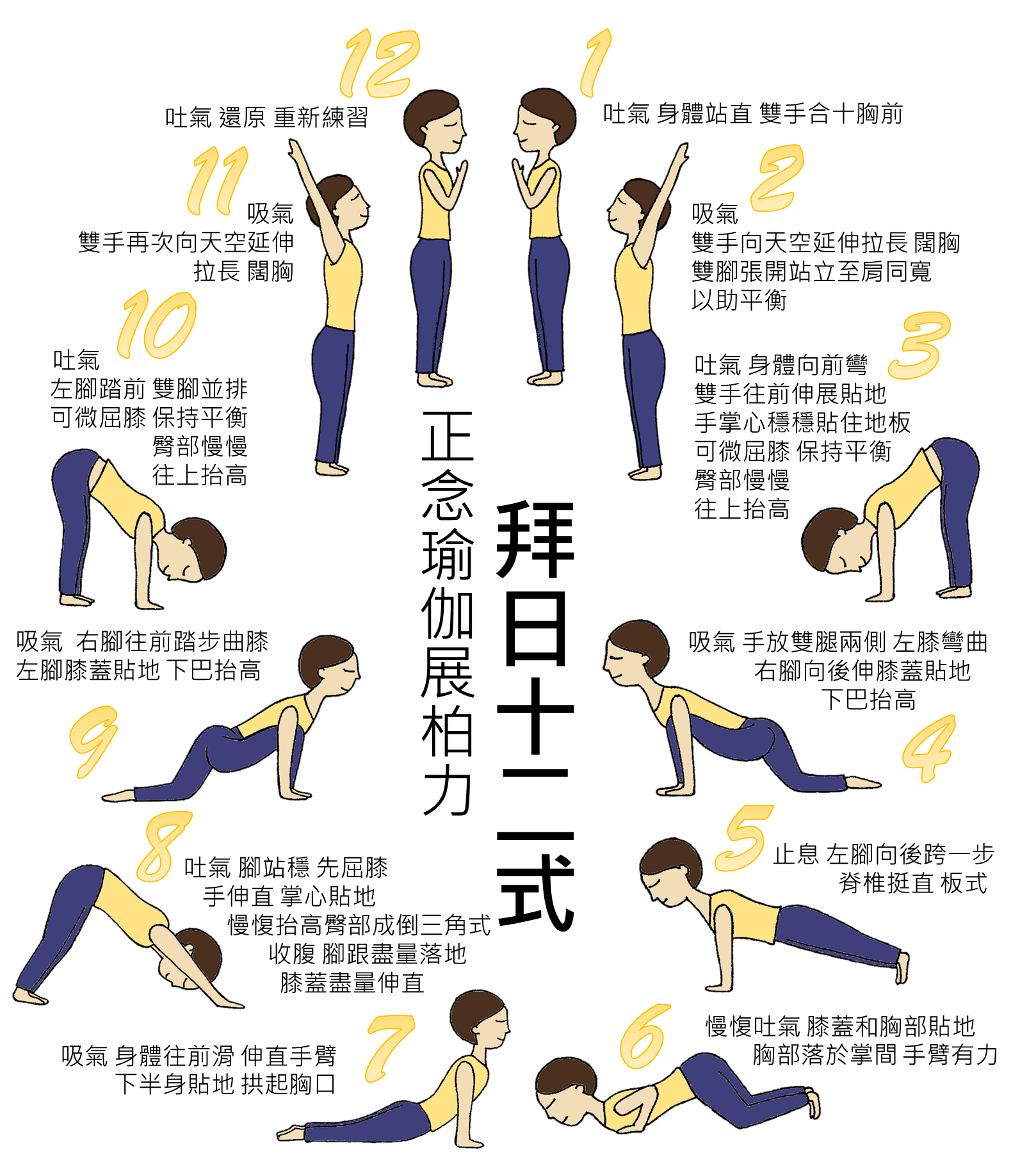
**

**伸展運動**


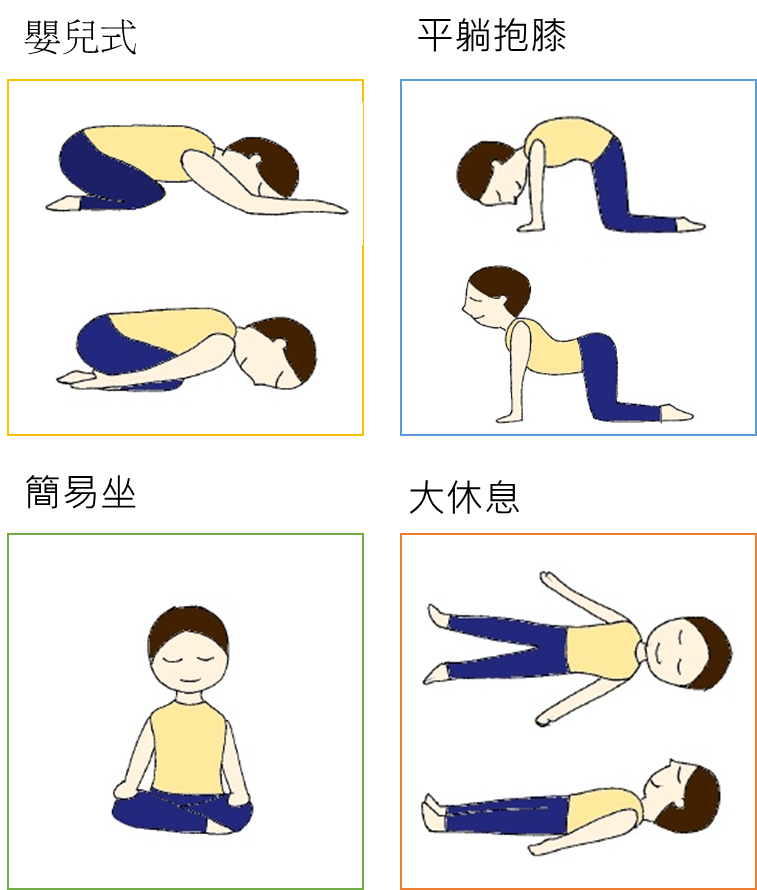


**靜觀練習**

- 1. 先坐在椅子，找一個舒適的姿勢安頓下來。
  2. 讓背部保持垂直，請勿靠著椅背，雙腳分開踏著地，保持舒適的坐姿，慢慢閉上眼睛。
  3. 感覺身體跟地板，椅子，坐墊接觸的地方。用一兩分鐘就如在身體掃描中，留心一下這些感覺。
  4. 現在將注意力移到下腹，感覺一下一吸一呼之間，下腹的變化。開始時，可以把手放在下腹，覺察手部接觸到的地方的變化。慢慢察覺到這些地方的感覺後，就可放開雙手，繼續留心肚皮的感覺。
  5. 留心吸氣時，肚皮微微張開的感覺，呼氣時肚皮微微放鬆落下。盡量留心整個歷程。吸氣時，空氣進入身體，呼氣時，空氣離開身體，下腹感覺如何變化，留心吸氣與呼氣之間的空隙。毋需用任何方法控制呼吸，盡量順其自然，對呼吸如此，對其他經驗亦如此。毋需改正什麼，追尋什麼。
  6. 很快你就會分心，由觀察下腹轉到其他念頭，如作白日夢，想起生活中的一些事情等等。但不要緊，這不是錯誤或失敗，分心是很自然的。當你留心到你分了心，輕輕的恭喜自己──你又已回到當下！溫柔的把注意力帶回到下腹變化中的感覺上，重新留心一吸一呼。
  7. 雖然我們會不斷的分心，但無論分心多少次，每次一察覺到分了心，就可以恭喜自己，又回到當下了！重新留心呼吸，下腹的變化。盡量帶著慈愛的去覺察，每次分心，都可視之為一個機會，培養耐性和好奇，善待自己。
  8. 練習最少十五分鐘，不時提醒自己，旨在留心每一刻的體驗，穩住於呼吸，活於此時此地。每次發現自己分了心，又把注意帶回下腹，覺察一吸一呼。這個練習旨在提高我們的覺察力。很多時候，我們未能如實的觀察當下發生的事，因為我們慣於立即斷定眼前的事物未夠好、未如意、不應該如此、或未達到心中要求。一經定斷，種種念頭情緒就接踵而來：埋怨、失望、想改變事實等等，把我們不知不覺帶入慣常的思路歷程，我們再覺察不到此刻發生的事物，亦再沒有自由去細慮及選擇處理應變的方法。

靜觀練習提示

1. 無論經歷如何，例如練習中睡著了、分心了、老想著其他事情、把注意力放到錯的身體部份，或毫無感覺都不緊要，堅持下去！這些都是你此刻的感覺，覺察它們。
2. 如你很容易分心，請視這些念頭如過客，又重新溫柔地把注意力帶回身體掃描上。
3. 放開「成」、「敗」、「好」、「壞」的念頭。這不是比賽，也不是要拼勁取得的技能。只要持之有恆，帶著開放好奇的心貫徹始終定時練習使好。
4. 放開對任何身體掃描的期望：想像它如一粒你種下的種子。你愈刻意去轉動干擾它，它就愈難成長。因此，做身體掃描時，只需讓它有理想的條件──安靜的環境，定時經常的練習，如此而已。你愈造作影響它，它愈難發揮功效。
5. 每一刻，嘗試保持開放的態度：「是的，此刻事物就是這樣。」如果你嘗試揮去不快的念頭、情緒、或身體感受，這些不快的情緒只會令你分心，忘了正在做的練習。請記著，無需強求，活在此刻，一切作如是觀。貫徹不捨。
